# Three new sorediate species of *Lecanora* sensu lato (*Ascomycota*, *Lecanoraceae*) from Anhui Province, China

**DOI:** 10.3897/mycokeys.136.191950

**Published:** 2026-07-15

**Authors:** Yi-Shan Feng, Li-Juan Li, Yan-Yun Zhang

**Affiliations:** 1 Key Laboratory of Biodiversity Conservation and Ecological Security in the Yangtze River Basin of Anhui Province, College of Life Sciences, Anhui Normal University, 241000 Wuhu, China Goethe University Frankfurt Frankfurt am Main Germany; 2 Goethe University Frankfurt, 60438, Frankfurt am Main, Germany Key Laboratory of Biodiversity Conservation and Ecological Security in the Yangtze River Basin of Anhui Province, College of Life Sciences, Anhui Normal University Wuhu China; 3 Senckenberg Research Institute and Natural History Museum, 60325, Frankfurt am Main, Germany Senckenberg Research Institute and Natural History Museum Frankfurt am Main Germany

**Keywords:** Eastern China, sorediate lichens, taxonomy, *
Verseghya
*

## Abstract

In this study, three new sorediate species, *Lecanora apicifarinacea* Y. S. Feng & Y. Y. Zhang, *L. pseudolayana* Y. S. Feng & Y. Y. Zhang and *Verseghya megasorediata* Y. S. Feng & Y. Y. Zhang from Anhui Province, China, are described and illustrated based on an integrated analysis of morphological, chemical and molecular data. Phylogenetically, *L. apicifarinacea* and *L. pseudolayana* are nested within the *L. subfusca* group. *Lecanora apicifarinacea* is characterized by the raised and erumpent soralia that aggregated into irregular patches, and the presence of atranorin and usnic acid. *L. pseudolayana* is characterized by an amphithecium with small crystals, an egranulose epihymenium, marginally small and dispersed but centrally large and aggregated soralia, and the presence of atranorin and zeorin. *Verseghya megasorediata* is nested within the genus *Verseghya* and is sister to *V. thysanophora*. This species is distinguished by its leprose thallus with a white fibrous prothallus, coarse and large soredia, [22.5]–(27.8)–38.9–(50.0)–[87.5] µm in diameter (n = 155), and the presence of usnic acid. In addition, *Verseghya klarae* is reduced to synonymy with *V. thysanophora*, based on their morphological similarity, identical secondary metabolites, and the lack of phylogenetic distance. A key to the *Lecanora* sensu lato species in Anhui Province is provided.

## Introduction

Historically, *Lecanora* sensu lato encompassed most species (ca. 1000 worldwide) of the family *Lecanoraceae* (Yakovchenko et al. 2019; Santos et al. 2023; Ivanovich-Hichins et al. 2025a), of which 109 species have been recorded in China (Wei 2020). For a long time, numerous lichenologists have attempted to partition this heterogeneous genus into more natural units, resulting in the segregation of many lineages into distinct genera within *Lecanoraceae*, e.g. *Flavonora* Mazur, Malíček & Śliwa, *Glaucomaria* M. Choisy, *Lecanoropsis* M. Choisy, *Myriolecis* Clem., *Protoparmeliopsis* M. Choisy, *Pulvinora* Davydov, *Straminella* M. Choisy, *Verseghya* S.Y. Kondr., Lőkös & Hur, and *Zeora* Fr. (Fries 1825; Choisy 1929, 1949; Kondratyuk et al. 2016, 2019; Zhao et al. 2016; Davydov et al. 2021; Ivanovich-Hichins et al. 2025a, 2025b; Mazur et al. 2025). Nevertheless, *Lecanora* s. lat. remains a paraphyletic group, including *Lecanora* s. str. (= the *L. subfusca* group), *L. albella-subcarnea* group, *L. fuscescens* group, *L. intumescens* group, *L. marginata* group, and *L. polytropa* group, which underscores the need for continued, in-depth morphological and phylogenetic investigation to clarify their taxonomic positions (Brodo et al. 2019; Bungartz et al. 2020; Øvstedal et al. 2020; Zhang et al. 2022; Arup et al. 2023; Ivanovich-Hichins et al. 2025b).

Members of the genus *Lecanora* s. lat. as currently recognized are predominantly sexually reproducing, but vegetative reproduction via soredia is also common (Malíček et al. 2017). Over the past five years, our repeated field surveys in Anhui Province, China have collected numerous sorediate *Lecanora* specimens, which we refer to the *L. subfusca* group and to the genus *Verseghya*.

To date, ca. 43 sorediate species of the *Lecanora subfusca* group have been reported worldwide (Tønsberg 1992; Ryan 2004; Zduńczyk and Kukwa 2014; Malíček et al. 2017; Asher and Lendemer 2018; Cannon et al. 2022; Park et al. 2023; Watts et al. 2024). Based on morphological and chemical characteristics, Brodo et al. (1994) summarized the sorediate, saxicolous species of the *L. subfusca* group in Europe. Subsequently, the study of Malíček et al. (2017) focused on corticolous, atranorin-containing sorediate *Lecanora* species across the continent, outlined the phylogenetic positions of most species based on nrITS and mtSSU sequences, and revealed that some fertile specimens cannot be unambiguously identified without DNA sequence data due to high intraspecific variability and limited diagnostic phenotypic characters. Park et al. (2023) provided an identification key for sorediate *Lecanora* species in South Korea and described a new species, *L. neobarkmaniana* J. S. Park & S. O. Oh, based on its fimbriate margin, patchy soralia and molecular difference. Watts et al. (2024) provided a key to sorediate *Lecanora* species in western North America, noting that some sterile species form pairs with fertile taxa.

The genus *Verseghya* was established by S.Y. Kondratyuk, Laszlo Lőkös and Jae-Seoun Hur, which is characterized by a leprose thallus, a fibrous prothallus and the production of usnic acid (Kondratyuk et al. 2016, 2019). Although the genus consistently forms a well-supported monophyletic clade within *Lecanoraceae*, its precise phylogenetic position remains uncertain (Kondratyuk et al. 2016; Guzow-Krzemińska et al. 2017; Malíček et al. 2017; Watts et al. 2024). Currently, only two species are recognized in this genus: *V. klarae* S.Y. Kondr., Lőkös & Hur and *V. thysanophora* (R.C. Harris) S.Y. Kondr., Lőkös, Farkas & Hur. *V. klarae* is known only from South Korea (Kondratyuk et al. 2019), whereas *V. thysanophora* has a broader distribution, with records from East Asia, Europe and North America (Harris et al. 2000; Han et al. 2011; Guzow-Krzemińska et al. 2017; Malíček et al. 2017; Yakovchenko et al. 2018; Denchev et al. 2022).

In Anhui Province, China, which is located in a temperate-subtropical transitional zone, 15 species of *Lecanora* have previously been recorded, including the sorediate species *L. layana* Lendemer and *Verseghya thysanophora* (Feng et al. 2026). *Lecanora layana* is consistently sterile (Lendemer 2015; Feng et al. 2026), whereas *Verseghya thysanophora*, although sometimes fertile in other regions, has not been found with apothecia in Anhui Province (Harris et al. 2000; Han et al. 2011). Additionally, while *L. allophana* (Ach.) Nyl. is occasionally reported as sorediate (Ryan 2004), no sorediate specimens have been documented from the province (Lü et al. 2008). Against this background, our recent fieldwork in the province yielded additional sorediate *Lecanora* specimens that could not be assigned to any known species. Based on integrated morphological, chemical, and molecular phylogenetic evidence, we herein describe three new sorediate species of *Lecanora* s. lat. from Anhui Province, China. This discovery not only expands the known diversity of *Lecanora* s. lat. in the region but also provides new insights into the evolutionary significance of soredia within this genus.

## Materials and methods

### Morphology and chemistry

The specimens in this study are deposited in the Botany Herbarium of Anhui Normal University (**ANUB**), Senckenberg Gesellschaft für Naturforschung: Senckenberg Forschungsinstitut und Naturmuseum (**FR**), and Herbarium Mycologicum Academiae Sinicae-Lichenes (**HMAS-L**).

External morphological features were observed, measured, and described using an OLYMPUS SZ61TR (Hachioji, Japan) stereomicroscope. Key characteristics were photographed with a Mingmei MS60-2 (Guangzhou, China) camera. Thin sections of the thallus and apothecia were prepared manually using a razor blade, mounted in water, and examined under an OLYMPUS BX43 (Hachioji, Japan) compound microscope to observe anatomical structures. Photomicrographs were taken using a Mingmei MDX10 (Guangzhou, China) camera. Soredia and spore measurement data is presented as: [minimum]–(x̄ – SD) – x̄ – (x̄ + SD)–[maximum], where x̄ is the arithmetic mean, SD is the standard deviation (values were rounded to the nearest 0.5 µm), followed by the number of measurements (n) (Lendemer 2015; Li et al. 2023b). Crystals in apothecia were observed in polarized light (POL), their solubility was studied in 20% nitric acid (20% HNO_3_ = “N”) and 10% potassium hydroxide (10% KOH = “K”), N-sol/K-sol means crystals dissolved, N-insol/K-insol means they did not dissolve.

Spot tests were conducted using K, C [10% calcium hypochlorite Ca(ClO)_2_], and KC (K followed by C). Thin-layer chromatography (TLC) analyses were undertaken in solvent system C, following standard methods (Orange et al. 2001).

### DNA extraction, PCR and sequencing

Soredia or apothecia from dry specimens were taken to extract genomic DNA, using the Chelex® 100 Resin (Bio-Rad, Hercules, CA, USA) method (Ferencová et al. 2017) according to the manufacturer’s instructions. For the PCR amplification of the nrITS region we used primers ITS1F (5' CTTGGTCATTTAGAGGAAGTAA 3') and ITS4a (5' CGCCGTTACTGGGGCAATCCCTG 3') (Gardes and Bruns 1993; Larena et al. 1999), for the nrLSU region primers LR0R (5' GTACCCGCT GAACTTAAGC 3') and LR5 (5' ATCCTGAGGGAAACTTC 3') (Vilgalys and Hester 1990; Rehner and Samuels 1994), and for the mtSSU region primers mrSSU1 (5' AGCAGTGAGGAATATTGGTC 3') and mrSSU3R (5' ATGTGGCACGTCTATAGCCC 3') (Zoller et al. 2007) or 16F (5' CAGCAA CTTGGAGGAATG 3') and 972R (5' ATGATGACTTGTCTT AGTCC 3') (Li et al. 2023a). The PCR reaction mix included (in the total volume of 25 µl): 9.5 μl dd H_2_O, 12.5 μl 2 × Trio Taq Master Mix (Monad, Hefei, Anhui, China), 1 μl of each primer, and 1 μl of DNA. The PCR amplification conditions for nrITS and nrLSU were: initial denaturation at 94 °C for 5 min; 35 cycles of denaturation at 94 °C for 15 s, annealing at 53 °C for 15 s, and extension at 72 °C for 1 min; final extension at 72 °C for 10 min; hold at 4 °C. The mtSSU was amplified using the following procedure: initial denaturation at 94 °C for 5 min, followed by 4 cycles of 94 °C for 30 s, 54 °C for 30 s and 72 °C for 1 min, 30 cycles of 94 °C for 30 s, 50 °C for 30 s and 72 °C for 1 min and a final extension at 72 °C for 10 min; hold at 4 °C (Zhang et al. 2024). PCR products were sent to General Biosystems (Chuzhou, Anhui, China) or Macrogen Europe (Amsterdam, The Netherlands) for sequencing using the amplification primers.

### Phylogenetic analyses

Using the NCBI online BLAST tool (https://blast.ncbi.nlm.nih.gov/Blast.cgi), we analyzed the raw sequences to ascertain that the DNA belonged to lichenized fungi, made a preliminary assessment of their taxonomic status, and downloaded the most similar homologous sequences (Table 1). Geneious 2025.0.2 was used to assemble and edit the raw sequences and generate a single matrix for nrITS, nrLSU and mtSSU. Each sequence was aligned using the online MAFFT service (https://mafft.cbrc.jb/alignment/server/index.html) with the E-INS-I strategy (Katoh and Standley 2013; Katoh et al. 2019) under default parameters. The alignments were checked in MEGA v. 7 (Kumar et al. 2016) and minor misalignments were manually adjusted. Before concatenating the single-gene datasets, we tested for potential incongruity using the online version of IQ-TREE (Trifinopoulos et al. 2016, https://iqtree.cibiv.univie.ac.at/) with 1000 ultrafast bootstrap replicates. No well supported conflict was detected, as no incongruent nodes reached UFBoot ≥ 95 (Minh et al. 2013).

**Table 1.** Specimens used for the phylogenetic analyses with the corresponding voucher information and GenBank accession numbers for nrITS, nrLSU and mtSSU sequences. Newly-obtained sequences are in bold font. “na” indicates that there is no sequence available.

**Species name**	**Country**	**Voucher specimens**	**GenBank accession number**		
			**nrITS**	**nrLSU**	**mtSSU**
*Flavonora inversa*	Bolivia	Flakus 29124 (KRAM)	OL604044	OL663903	OL604124
*Lecanora allophana*	Finland	Malíček 9491 (JM)	KY548051	na	KY502416
*L. anhuiensis* 1	China: Anhui	Ren 20200748 (HMAS-L)	OR098679	OR096274	OR096240
*L. anhuiensis* 2	China: Anhui	Ren 20200731 (HMAS-L)	OR098678	OR096275	OR096242
*L. apicifarinacea*	China: Anhui	ZYY 22-1025 (ANUB)	**PX843658**	na	na
*L. arachnoidea*	Czech Republic	Vondrák 24028 (PRA)	OL457932	na	OK465506
*L. baekdudaeganensis* 1	China: Anhui	YYJ 24-197 (ANUB)	PV505370	na	na
*L. baekdudaeganensis* 2	South Korea	BDNA-L-0000065 (KBA)	NR_184912	na	MN879871
*L. barkmaniana*	Austria	Malíček 7353 (JM)	KT630246	na	KT630258
*L. campestris*	Portugal	Sipman 62850	MN586965	na	na
L. cenisia f. soredians	Czech Republic	Malíček 15774	OQ918726	na	OQ920112
*L. chloroleprosa*	USA	McCune 36469 (OSC)	MN906279	na	na
*L. crystalliniformis*	China	Wang et al. 17-56085 (KUN-L)	ON807163	na	ON807173
*L. elatinoides*	Australia	HTL 19992d (F)	JQ782709	na	JQ782669
*L. epanora*	Czech Republic	Malíček 17109 (PRA)	PV536059	na	PV536151
*L. epibryon*	USA	Leavitt 18-543 (BRY-C)	MZ243579	na	na
*L. exspersa*	Russia	Malíček 9629 (JM)	KY548057	na	KY502415
*L. farinaria*	Norway	Palice 20106 (PRA)	KY548042	na	na
*L. flavidomarginata* 1	Bolivia	Flakus 29951 (KRAM)	OL604056	OL663915	OL604135
*L. flavidomarginata* 2	Bolivia	Flakus 28943 (KRAM)	OL603996	na	OL604077
*L. fulvastra*	Japan	Sakata 3591 (CBM)	LC269720	na	na
*L. helva*	Thailand	Papong 6444 (F)	JQ782716	na	JQ782679
*L. impudens*	Switzerland	LIFU 088-16	KX132996	na	na
*L. imshaugii*	USA	HTL 19273b (F)	JQ782717	na	JQ782681
*L. kenyana* 1	Kenya	Kirika 1179 (F)	NR_120112	na	na
*L. kenyana* 2	Kenya	Kirika 1179 (F)	JQ900618	na	JQ900616
*L. layana* 1	USA	Lendemer 38131 (NY)	NR_158472	na	KR094858
*L. layana* 2	China: Anhui	ZYY 24-1072 (ANUB)	PV505355	na	na
*L. leproplaca*	Kenya	HTL 19558m (F)	JQ782718	na	JQ782683
*L. loekoesii*	China: Heilongjiang	Wei et al. HLJ 201400311 (HMAS-L)	OR098694	na	OR096237
*L. markjohnstonii*	USA	E.A. Tripp 6296 & J.C. Lendemer (COLO)	MH887500	na	na
*L. neobarkmaniana* 1	South Korea	KHL 0031345 (KH)	OP090560	na	OP099444
*L. neobarkmaniana* 2	South Korea	KHL 0035545 (KH)	OP090561	na	OP099445
*L. norvegica*	Czech Republic	Vondrak 18718 (PRA)	OQ717446	na	na
*L. orientoafricana*	Kenya	Kirika 2205 (F)	NR_120113	na	JQ900617
*L. pseudojaponica* 1	China: Anhui	Yao 20200919 (HMAS-L)	OR098682	na	OR096243
*L. pseudojaponica* 2	China: Anhui	Yao 20200932 (HMAS-L)	OR098687	OR096276	OR096246
*L. pseudolayana* 1	China: Anhui	Zhang 20200510 (HMAS-L)	**PZ157812**	na	**PZ157465**
*L. pseudolayana* 2	China: Anhui	ZYY 24-1511 (ANUB)	**PX843653**	**PX843667**	**PX853214**
*L. pseudolayana* 3	China: Anhui	ZYY 24-1580 (ANUB)	**PX843654**	na	na
*L. pseudolayana* 4	China: Anhui	ZYY 24-1505 (ANUB)	**PX843655**	na	na
*L. pseudolayana* 5	China: Anhui	ZYY 24-1115 (ANUB)	**PX843656**	na	na
*L. pseudolayana* 6	China: Anhui	ZYY 24-1549 (ANUB)	**PX843657**	na	na
*L. substerilis*	Slovakia	Vondrák 12294 (PRA)	KT630243	na	KT630254
*L. ussuriensis*	South Korea	Kondratyuk & Lőkös 040227 (KoLRI)	MK672832	na	na
*L. variolascens*	Austria	Malíček 8422 (JM)	KY548038	na	KY502445
*L. zeorina*	China	Wang et al. 19-63051 (KUN-L)	ON807167	na	ON807168
*Protoparmelia badia*	USA	Fryday 8575	KY066254	KY066280	KY012807
*P. picea*	Norway	Haugan 9612 (O)	KF562194	KF562186	na
*Pulvinora cavicola*	Bolivia	Flakus 29567 (KRAM)	OL604040	OL663900	OL604119
*Straminella conizaeoides*	Czech Republic	244_CZ	MT938947	na	MT939177
*Verseghya klarae* 1	South Korea	Lőkös & Kondratyuk 034123 (KoLRI)	MK672855	na	MK693697
*V. klarae* 2	South Korea	Lőkös & Kondratyuk 034122 (KoLRI)	MK672854	na	MK693696
*V. klarae* 3	South Korea	Kondratyuk & Lokos 034088 (KoLRI)	MK672853	na	MK693695
*V. megasorediata* 1	China: Anhui	ZYY 24-1084 (ANUB)	**PX843642**	**PX843659**	**PX853206**
*V. megasorediata* 2	China: Anhui	ZYY 24-1392 (ANUB)	**PX843643**	na	na
*V. megasorediata* 3	China: Anhui	ZYY 24-1405 (ANUB)	**PX843644**	na	na
*V. megasorediata* 4	China: Anhui	ZYY 24-1422 (ANUB)	**PX843645**	**PX843660**	**PX853207**
*V. megasorediata* 5	China: Anhui	YYJ 24-204 (ANUB)	**PX843646**	**PX843661**	**PX853208**
*V. megasorediata* 6	China: Anhui	ZYY 24-1506 (ANUB)	**PX843647**	na	na
*V. megasorediata* 7	China: Anhui	ZYY 24-1445 (ANUB)	**PX843648**	**PX843662**	**PX853209**
*V. megasorediata* 8	China: Anhui	ZYY 21-83 (ANUB)	**PX843649**	**PX843663**	**PX853210**
*V. megasorediata* 9	China: Anhui	ZYY 24-1126 (ANUB)	**PX843650**	**PX843664**	**PX853211**
*V. megasorediata* 10	China: Anhui	ZYY 24-1473 (ANUB)	**PX843651**	**PX843665**	**PX853212**
*V. megasorediata* 11	China: Anhui	ZYY 24-1658 (ANUB)	**PX843652**	**PX843666**	**PX853213**
*Verseghya* sp.	Russia	Malíček et al. 10608 (JM)	MG076967	na	na
*V. thysanophora* 1	Czech Republic	Vondrak 24836 (PRA)	OQ717906	na	OQ646277
*V. thysanophora* 2	Czech Republic	Vondrak 23514 (PRA)	OQ717905	na	OQ646278
*V. thysanophora* 3	Poland	17775	MN387223	na	na
*V. thysanophora* 4	Poland	17188	MN387222	na	na
*V. thysanophora* 5	Poland	Łubek 17245 (KTC)	KY586042	na	na
*V. thysanophora* 6	South Korea	CP 14122 (FR)	**PZ157808**	na	**PZ157461**
*V. thysanophora* 7	Japan	—	LC700475	na	na
*V. thysanophora* 8	Japan	CP 15197 (FR)	**PZ157809**	**PZ151436**	**PZ157462**
*V. thysanophora* 9	Japan	CP 15200 (FR)	**PZ157810**	na	**PZ157463**
*V. thysanophora* 10	Japan	CP 15462 (FR)	**PZ157811**	**PZ151437**	**PZ157464**
*V. thysanophora* 11	USA	Lendemer 13179 (NY)	Na	na	KC184000
*V. thysanophora* 12	USA	Lendemer 16933 (NY)	Na	na	KC184024
*V. thysanophora* 13	China: Anhui	ZYY 24-1693 (ANUB)	**PX843640**	na	na
*V. thysanophora* 14	China: Anhui	ZYY 24-1612 (ANUB)	**PX843641**	na	na
*Zeora compallens*	Czech Republic	Palice 32857 (PRA)	OQ717435	na	na
*Z. confusa*	U.K.	B.J.&A.M.Coppinss.n. (E)	GU480120	na	na
*Z. expallens*	Finland	Malíček 9510 (JM)	MG076969	na	na
*Z. flavoleprosa*	Czech Republic	Malíček 13901	MW979430	na	MW979425
*Z. helmutii*	Australia	Kantvilas 19506 (MA)	NR_161059	na	na
*Z. orosthea*	Czech Republic	Vondrák 24522 (PRA)	OK332974	na	OK465589
*Z. stanislai*	Poland	Łubek 13009 (UGDA)	KY586046	na	na
*Z. strobilina*	Spain	Pérez-Ortega 5270 & Prats 19510 (MA)	MG973235	na	na

The concatenated nrITS-nrLSU-mtSSU sequences were obtained via the Concatenate Sequence function in PhyloSuite v1.2.3 (Xiang et al. 2023), the alignment comprised 161 sequences, including 112 obtained from GenBank and 49 newly generated in this study (Table 1). This dataset encompasses the majority of the available sequences of sorediate species within *Lecanora*, as well as the ones from closely related species of the new species. According to the phylogenetic framework of *Lecanoraceae* (Zhao et al. 2016), *Protoparmelia picea* (Dicks.) Hafellner and *P. badia* (Hoffm.) Hafellner were selected as the outgroup for our phylogenetic analysis. ModelFinder (Kalyaanamoorthy et al. 2017) was used to estimate the best schemes under the Bayesian Information Criterion (BIC) and nucleotide substitution models. The best schemes and selected models are shown in Table 2.

**Table 2.** The best schemes and nucleotide substitution models selected by ModelFinder for the concatenated 3-gene dataset.

**Partition**	**Regions**	**Positions**	**Model for IQ-TREE**	**Model for Bayes**
Partition 1	ITS1	1–284	TIM2+F+G4	GTR+F+G4
Partition 2	5.8S	285–455	TNe+R2	K2P+I+G4
Partition 3	ITS2	456–554	TN+F+G4	HKY+F+G4
Partition 4	nrLSU	555–1265	TNe+R2	K2P+I+G4
Partition 5	mtSSU	1266–1877	TPM2u+F+I+G4	HKY+F+I+G4

**Figure 1.**
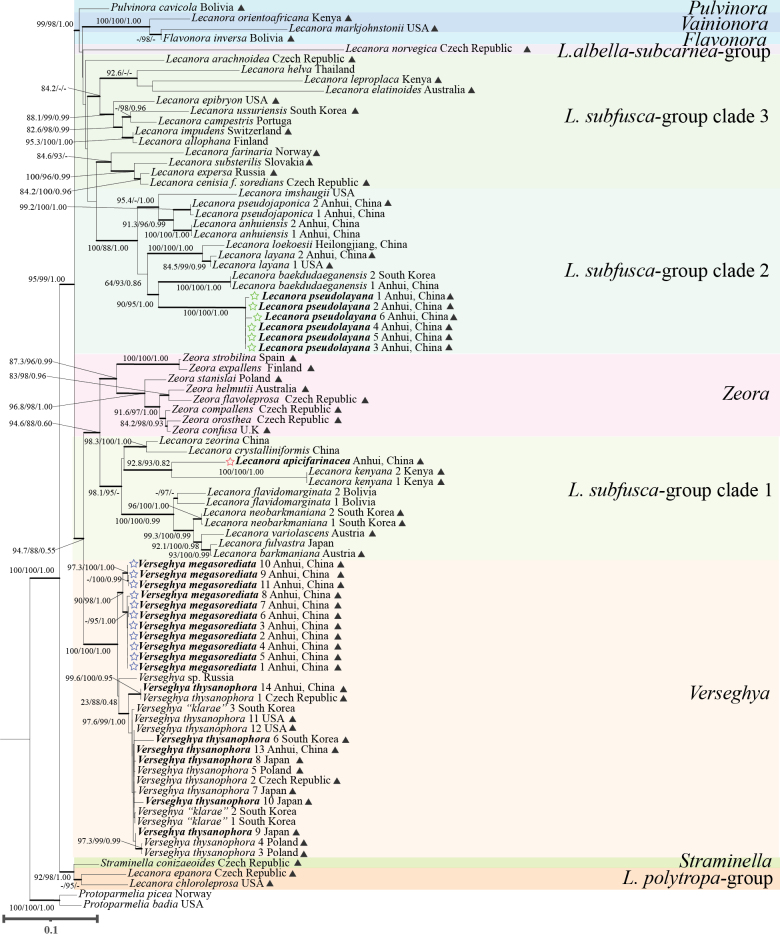
Maximum Likelihood (ML) phylogenetic tree, based on the concatenated nrITS, nrLSU and mtSSU dataset. SH-aLRT support ≥ 80% /Ultrafast bootstrap support ≥ 95% /Bayesian posterior probabilities ≥ 0.90 are displayed along the branches of the tree. Branches that attained the support from at least one of the three inference methods are shown in bold. Newly-generated sequences are indicated in bold. The three new taxa are marked by pentagrams. The sorediate species are marked by triangles.

Maximum likelihood (ML) analyses and Bayesian inference (BI) were carried out using PhyloSuite v.1.2.3 (Xiang et al. 2023). ML tree analyses were performed using IQ-TREE (Nguyen et al. 2015; Trifinopoulos et al. 2016). Statistical support values were estimated using ultrafast bootstrapping with 1000 replicates, while keeping all other parameters at their default settings. Bayesian tree inference was carried out by MrBayes 3.2 (Ronquist et al. 2012). Four chains including three heated and one cold were used, with an initial run of 2 million generations. Samples were taken every 1,000^th^ generation, and the burn-in was set to 0.25 (indicating that the first 25% of samples were discarded). Bayesian analysis was considered converged when the Average Standard Deviation of Split Frequencies (ASDSF) value was less than 0.01. All other parameters were set to default values. Support values were shown on the branches when the Shimodaira-Hasegawa-like approximate likelihood ratio test (SH-aLRT) was ≥ 80%, the ultrafast bootstrap approximation (UFBoot) was ≥ 95% (both calculated from the same IQ-tree run) and Bayesian posterior probabilities (BPP) were ≥ 0.90. The phylogenetic tree was visualized using FigTree v.1.4.4 and subsequently refined and edited in Adobe Illustrator 2020 SP. The ML tree is presented in the main text (Fig. 1).

## Results and discussion

The three-locus phylogenetic tree (Fig. 1) shows that the newly described species *L. apicifarinacea*, is sister to *L. kenyana* Kirika & Lumbsch, a sorediate species recently described from Kenya, which belongs to *L. subfusca* group clade 1 (Kirika et al. 2012). Another newly described species, *Lecanora pseudolayana*, together with three previously known species, *L. baekdudaeganensis* B.G. Lee & Hur, *L. layana* and *L. loekoesii* L. Lü, Y. Joshi & Hur, forms a subclade belonging to the *L. subfusca* group clade 2. Within this subclade, *L. pseudolayana* is strongly supported as sister to *L. baekdudaeganensis* (90/95/1.00), and clusters with a subclade formed by *L. layana* and *L. loekoesii*. Morphologically, *L. pseudolayana* and *L. layana* are sorediate (Lendemer 2015), whereas *L. baekdudaeganensis* and *L. loekoesii* are esorediate (Wang et al. 2013; Lee and Hur 2020).

The genus *Verseghya* was recovered as monophyletic with strong support (100/100/1.00) in all analyses, consistent with previous work (Kondratyuk et al. 2019), and currently comprises two described species, *V. klarae* and *V. thysanophora*. However, specimens identified under these two names from diverse geographic origins (China, the Czech Republic, Japan, Poland, South Korea, and the United States) formed a cohesive and well-supported clade (97.6/99/1.00) (Fig. 1), suggesting that *V. klarae* and *V. thysanophora* are conspecific. Meanwhile, the newly proposed species *V. megasorediata* forms a well-supported monophyletic clade (90/98/1.00) and is resolved as sister to *V. thysanophora*.

### Taxonomy

#### Lecanora apicifarinacea

Taxon classificationFungiLecanoralesLecanoraceae

Y. S. Feng & Y. Y. Zhang sp. nov.

4E6BC8CF-36EB-5020-BB80-DDEDEE1B5E4C

Fungal Names: FN 573732

Fig. 2A–D

##### Etymology.

The epithet refers to the raised, apically erumpent to farinose soralia of this species.

**Figure 2.**
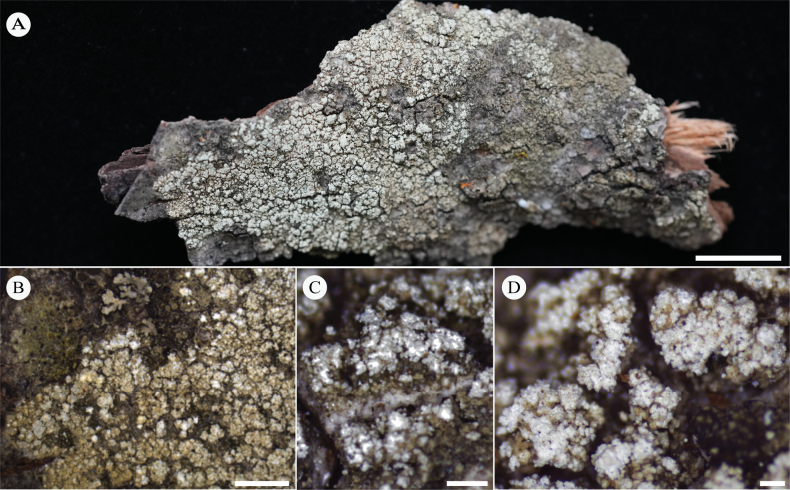
*Lecanora apicifarinacea*: **A**. Lichen thallus, habit; **B**. Irregular and diffuse thallus margin; **C**. Soralia; **D**. Soralia apically erumpent to white farinose. Scale bars: 1 cm (**A**); 2 mm (**B**); 1 mm (**C**); 0.4 mm (**D**).

##### Type.

China • Anhui Prov., Qianshan City, Tianzhushan World Geopark, 30°44'42"N, 116°27'11"E, 1377 m alt., on bark, 21 September 2022, Yanyun Zhang 22-1025 (***holotype***-ANUB879, ***isotype***-FR).

##### Diagnosis.

Distinguished from other sorediate species of *Lecanora* by the diffuse thallus with an irregular outline, the uprising, erumpent soralia aggregating into irregular patches, and the presence of atranorin and usnic acid.

##### Description.

Thallus crustose, thin, areolate, outline irregular, diffuse; prothallus absent; surface gray-green; soralia raised, up to 0.53 mm in height, [0.10]–(0.17)–0.42–(0.68)–[1.0] mm in diameter (n = 35), usually aggregated into irregular patches and covering most of the thallus, grayish yellow-green, apically erumpent to white farinose; soredia fine, granular, non-corticate, simple and loosely aggregated, [20.0]–(21.9)–27.1–(32.3)–[42.5] µm in diameter (n = 69); apothecia and pycnidia unknown.

##### Chemistry.

Thallus K+ yellow, C-, KC+ yellow; containing atranorin and usnic acid.

##### Distribution and ecology.

The new species has been found on bark at 1377 m altitude in Tianzhushan Mountain, a montane forest area from China.

##### Notes.

*Lecanora kenyana* is closely related to the new species and also contains atranorin and usnic acid, but *L. kenyana* differs in its verrucose thallus and the rounded, concave soralia (Kirika et al. 2012). While the species *L. barkmaniana* Aptroot & Herk, *L. neobarkmaniana* and *L. variolascens* Nyl. are also sorediate and closely related to the new species, *L. barkmaniana* and *L. neobarkmaniana* can be distinguished by their punctiform soralia and the presence of zeorin, additionally *L. neobarkmaniana* is a saxicolous species (Aptroot and Van Herk 1999; Park et al. 2023); *L. variolascens* differs in its discrete soralia and the presence of zeorin (Malíček et al. 2017).

The soralia of the new species resemble those of *Lecanora compallens* Herk & Aptroot and *L. markjohnstonii*. However, *L. compallens* can be distinguished by its yellowish to slightly mint-green soredia, and its initially punctiform soralia, 0.1–0.3 mm in diameter, soon aggregating into irregular patches, as well as by the presence of zeorin and usnic acid (Van Herk and Aptroot 1999; Urbanavichene et al. 2018; Cannon et al. 2022). *Lecanora markjohnstonii* differs in occurring on rock rather than bark, and in containing 2’-O-methylperlatolic acid as an accessory compound alongside atranorin, instead of usnic acid (Stewart et al. 2018).

#### Lecanora pseudolayana

Taxon classificationFungiLecanoralesLecanoraceae

Y. S. Feng & Y. Y. Zhang sp. nov.

FE6C96BA-DAC5-579C-AA47-B0C94413A8DC

Fungal Names: FN 573738

Fig. 3A–J

##### Etymology.

The specific epithet refers to the morphological similarity of this species to *L. layana*.

**Figure 3.**
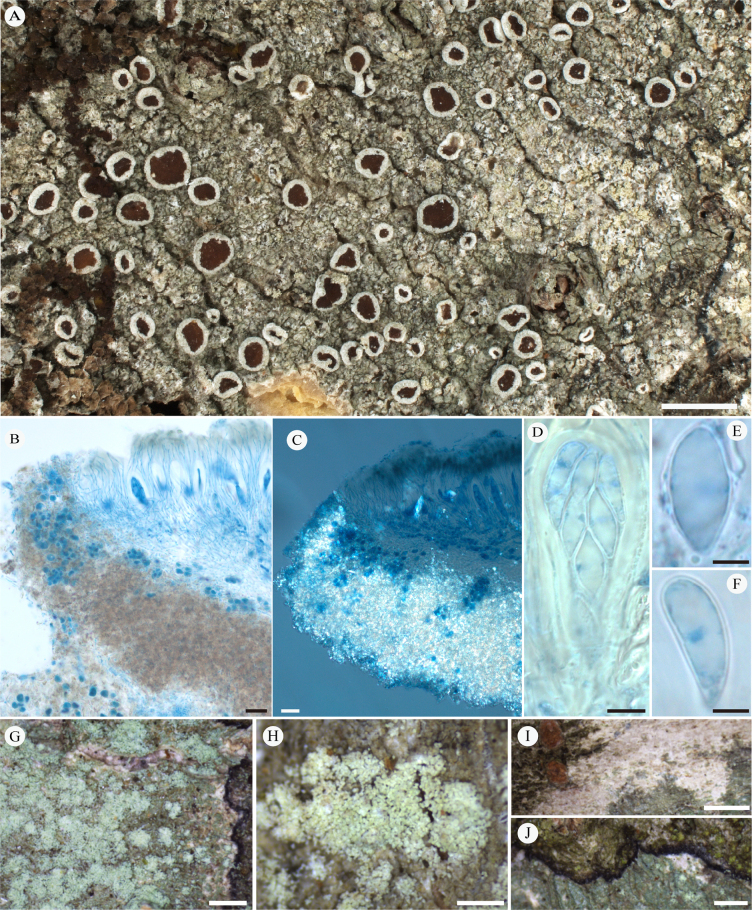
*Lecanora pseudolayana* (**A–F** are from HMAS-L-0147383, **G, H, J** are from ANUB 2240, **I** is from ANUB 1935): **A**. Lichen thallus and apothecia, habit; **B**. Vertical sections of apothecia (LCB); **C**. Vertical sections of apothecia in polarized light (LCB); **D**. 8-spored ascus; **E, F**. Ascospores; **G**. Thallus with soralia that are marginally small and dispersed, centrally large and aggregated; **H**. Soralia; **I, J**. Morphological variation in prothallus. Scale bars: 2 mm (**A, I**); 1 mm (**G, J**); 0.4 mm (**H**); 20 µm (**B, C**); 10 µm (**D, E, F**).

##### Type.

China • Anhui Prov., Lu’an City, Jinzhai County, Dabie Mountain, Tiantangzhai, 31°06'20"N, 115°46'15"E, 940 m alt., on bark, 12 October 2020, Jiarong Zhang 20200510, HMAS-L-0147383—***holotype***.

##### Diagnosis.

The species is characterized by the egranulose epihymenium, small amphithecial crystals that are insoluble in K but soluble in N, the blackish (N+ red) or white (N-) prothallus, the soralia that are marginally small and dispersed, centrally large and aggregated, and the presence of atranorin and zeorin.

##### Description.

Thallus crustose, thin, continuous or rimose to verrucose areolate when apothecia present; prothallus blackish (N+ red) when in contact with other lichen thalli or white (N-); surface gray-green, epruinose; soralia pale green to yellowish-green, becoming white-gray in the herbarium, rounded, convex, discrete in fertile specimens, marginally small and dispersed, [0.10]–(0.11)–0.20–(0.29)–[0.45] mm in diameter (n = 48), centrally large and aggregated, [0.15]–(0.19)–0.42–(0.65)–[1.0] mm in diameter (n = 49) when apothecia absent. Soredia fine, granular, non-corticate, simple and loosely aggregated, [15.0]–(19.8)–25.6–(31.5)–[42.5] µm in diameter (n = 198). Apothecia lecanorine, rounded, sparse to slightly aggregated, adnate, 0.5–1.2 mm in diameter; disc plane to concave, brown to reddish-brown, epruinose, margin persistent and prominent, entire to flexuous or slightly crenate, concolorous with thallus or paler to cream white, rather thick, 0.1–0.2 mm. Amphithecium with numerous algal cells, containing small crystals (POL+, K-insol, N-sol); cortex indistinct, interspersed with fine crystals (POL+, K-sol, N-insol); parathecium colorless, 10–15 µm wide, without crystals (POL-); epihymenium without crystals (POL-), with orange-brown to deep brown amorphous pigmentation not altered by K (or becoming slightly more dark brown), 10–20 µm high, orange intensifying in N; hymenium colorless, 80–100 µm high, distinctly delimited at the base; subhymenium colorless, 30–60 µm high; hypothecium colorless, composed of anastomosing hyphae, 20–50 µm high; paraphyses simple, more or less anastomosed, slender, c. 1.5 µm thick, tips slightly expanded up to 2.5 µm; asci clavate, *Lecanora*-type, 50–65 × 15–25 µm, 8-spored; ascospores simple, hyaline, narrowly or widely ellipsoid to subfusiform, dacriform or eye-shaped, [15.5]–(18.2)–20.1–(22.1)–[25.0] × [6.0]–(7.1)–7.9–(8.7)–[9.0] µm (n = 44), wall c. 0.5 µm. Pycnidia unknown.

##### Chemistry.

Thallus K+ yellow, C-, KC-; containing atranorin and zeorin.

##### Distribution and ecology.

This species occurs on bark of broadleaved trees and is known from Anhui Prov., in the south-eastern part of the Dabie Mountains, in the montane forests, at altitudes between 500 and 1219 m. The Dabie Mountains are located at the junction between Anhui, Hubei and Henan Provinces in China.

##### Notes.

*Lecanora baekdudaeganensis* is superficially similar to *L. pseudolayana*, but differs in its smaller ascospores and K-insoluble granules on the surface of the epihymenium (Lee and Hur 2020). *Lecanora imshaugii* Brodo differs in its smaller ascospores and the presence of hypoprotocetraric acid (Miyawaki 1988, 1994). *L. layana* can be readily distinguished by its soralia, which are immersed in the thallus and arise from the prothallus, rapidly eroding to become irregular, diffuse, and eventually confluent, and by its production of stictic acid (Lendemer 2015).

*Lecanora pseudolayana* can be easily misidentified as *L. appalachensis* Lendemer & R.C. Harris and *L. nothocaesiella* Lendemer & R.C. Harris. However, *L. appalachensis* has a white, fibrous prothallus and coarse soredia, [21]–(35)–48–(59)–[78] µm in diameter (vs. [15.0]–(19.8)–25.6–(31.5)–[42.5] µm in *L. pseudolayana*). The thallus of *L. nothocaesiella* is immersed in the substrate and soralia are diffuse, the prothallus is typically pale white to translucent in the center of the thallus (Lendemer et al. 2013). *Lecanora neobarkmaniana* is also a sorediate species that produces atranorin and zeorin; however, it is saxicolous and lacks a prothallus (Park et al. 2023).

##### Additional specimens examined.

China • Anhui Prov., Lu’an City, Jinzhai County, Dahuanglishuling, 31°10'10"N, 115°50'43"E, 797 m alt., on *Liquidambar formosana* bark, 7 August 2024, Yanyun Zhang and Xiaoying Wu 24-1549 (ANUB1935); • Kangwangzhai, 31°23'44"N, 115°23'04"E, 1219 m alt., on Lonicera japonica bark, 3 August 2024, Yanyun Zhang and Xiaoying Wu 24-1352 (ANUB1738); • Lijia Mountain, 31°9'22"N, 115°47'06"E, 867 m alt., on *Hovenia acerba* bark, 19 May 2024, Yanyun Zhang and Yujiao Yin 24-1115 (ANUB1494); • Wochuan Forest farm, 31°14'13"N, 115°40'07"E, 957 m alt., on *Liquidambar formosana* bark, 1 November 2024, Yanyun Zhang, Yujiao Yin and Yishan Feng 24-1580 (ANUB2240); • Xiaonanpeng, 31°16'01"N, 115°38'53"E, 500 m alt., on *Liquidambar formosana* bark, 6 August 2024, Yanyun Zhang and Xiaoying Wu 24-1505 (ANUB1891), 24-1511 (ANUB1897).

#### Verseghya megasorediata

Taxon classificationFungiPertusarialesPertusariaceae

Y. S. Feng & Y. Y. Zhang sp. nov.

A7A4A192-55CD-5B20-83ED-0EE6BC4E958B

Fungal Names: FN 573733

Fig. 4A–F

##### Etymology.

The epithet refers to its large soredia.

**Figure 4.**
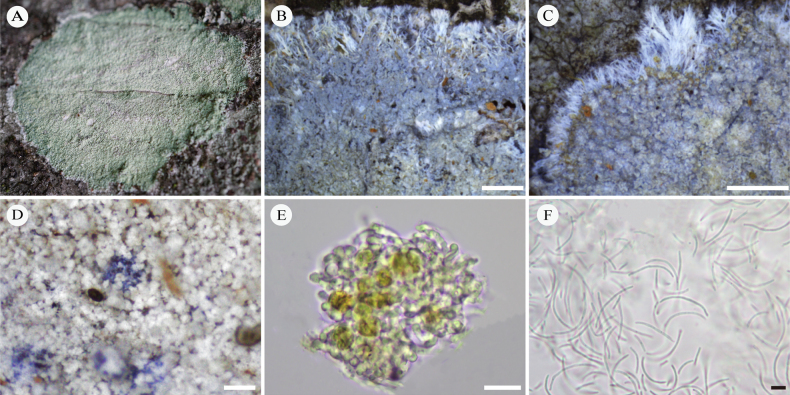
*Verseghya megasorediata* (**A, C, D, F** are from ANUB 1463, **B, E** are from ANUB 1505): **A**. Lichen thallus, habit; **B**. Leprose thallus; **C**. White fibrous prothallus; **D**. Pycnidia; **E**. Soredia mounted in water; **F**. Conidia. Scale bars: 2 mm (**B**); 1 mm (**C**); 0.4 mm (**D**); 20 µm (**E**); 5 µm (**F**).

##### Type.

China • Anhui Prov., Lu’an City, Jinzhai County, Tudiling, 31°15'31"N, 115°50'10"E, 846 m alt., on *Juglans regia* bark, 18 May 2024, Yanyun Zhang and Yujiao Yin 24-1084 (***holotype*-**ANUB1463, ***isotype***-FR).

##### Diagnosis.

Similar to *Verseghya thysanophora* in having a leprose thallus and white fibrous prothallus, but differing in its white fibrous prothallus never presenting blackish pigments, having coarse and large soredia, and not producing thysanophora unknowns as an accessory to ±atranorin, zeorin and usnic acid.

##### Description.

Thallus leprose, thin, continuous; prothallus white, fibrous, (0.05) 0.25–2.35 (3.25) mm wide, with hyphae aggregated into distinct radiating strands, hyphae ca. 2.5 µm wide; upper surface yellowish green, becoming white-gray in the herbarium; soredia green or white-gray, thick and covering the thallus, coarse, rounded, [22.5]–(27.8)–38.9–(50.0)–[87.5] µm in diameter (n = 155), non-corticate, often aggregated in elongate to rounded consoredia, up to 200 µm in diameter; pycnidia surrounded by soredia, uncommon, convex and globose, black, surface white arachnoid; conidia filiform, curved, 10–20 × ca. 0.5 µm; apothecia unknown.

##### Chemistry.

Thallus K- or K+ yellow, C-, KC+ weak yellow to yellow; containing ± atranorin, zeorin and usnic acid.

##### Distribution and ecology.

This species occurs on bark or trunks of broadleaved trees, or on rotting wood in montane forests at altitudes between 496 and 1354 m in Anhui Province.

##### Notes.

Although the new species *Verseghya megasorediata* is resolved into two highly supported subclades (Fig. 1), the short evolutionary distance between them and the absence of distinguishable morphological or chemical traits support treating samples from both subclades as conspecific.

*Verseghya megasorediata* closely resembles *V. thysanophora*. The latter can be differentiated, however, by its white, fibrous prothallus often presenting blackish pigments, its smaller soredia ([15.0]–(18.9)–24.0–(29.1)–[30.0] µm vs. [22.5]–(27.8)–38.9–(50.0)–[87.5] µm), and by the production of “thysanophora unknowns” in some specimens (Harris et al. 2000; Kondratyuk et al. 2016). The new species resembles *Lecanora markjohnstonii* and *L. arachnoidea* Vondrák, Malíček & Svoboda in sharing a sorediate thallus with a white, fibrous prothallus. However, *L. markjohnstonii* is distinguished by the presence of 2’-O-methylperlatolic acid and its saxicolous habit (Stewart et al. 2018), whereas *L. arachnoidea* differs in having a rimose-areolate thallus and the production of perlatolic acid (Vondrák et al. 2023). Another sorediate *Lecanora* species containing atranorin, zeorin, and usnic acid is *L. sibirica* Müll. Arg., which is characterized by a rimose-areolate or continuous to verrucose-areolate thallus and a blackish-brown prothallus (Lumbsch et al. 1997; Harada et al. 2004).

##### Additional specimens examined.

China • Anhui Prov., Lu’an City, Huoshan County, Mozitan Town, 31°6'34"N, 116°11'35"E, 1354 m alt., on bark, 1 April 2023, Lun Wang 23-23 (ANUB1062); • Taiyang Village, Baimajian Mountain, 31°7'03"N, 116°10'21"E, 1197 m alt., on rotting wood, 8 September 2021, Yanyun Zhang 21-83 (ANUB986). • Lu’an City, Jinzhai County, Ganghou Village, 31°17'11"N, 115°43'45"E, 958 m alt., on bark, 4 August 2024, Yanyun Zhang and Xiaoying Wu 24-1392 (ANUB1778); • Lijiashan Mountain, 31°9'18"N, 115°47'09"E, 910 m alt., on bark, 6 August 2024, Yujiao Yin and Xiangxiang Ge 24-204 (ANUB2157); • Mazongling, Tongtian Waterfall, 31°17'39"N, 115°41'05"E, 842–1045 m alt., on Broad leaved trunks, 5 August 2024, Yanyun Zhang and Xiaoying Wu 24-1473 (ANUB1859), 24-1445 (ANUB1831), 31°17'32"N, 115°41'07"E, 997 m alt., on *Cercis gigantean* bark, 2 November 2024, Yanyun Zhang, Yujiao Yin and Yishan Feng 24-1658 (ANUB2318); • Nanwudang, 31°9'09"N, 115°47'15"E, 1009 m alt., on bark, 19 May 2024, Yanyun Zhang and Yujiao Yin 24-1126 (ANUB1505); • Shengguajian Mountain, 31°16'57"N, 115°43'51"E, 1105–1197 m alt., on *Cyclobalanopsis glauca* trunk, 4 August 2024, Yanyun Zhang and Xiaoying Wu 24-1405 (ANUB1791), 24-1422 (ANUB1808); • Xiaonanpeng, 31°16'02"N, 115°38'52"E, 496 m alt., on *Liquidambar formosana* bark, 6 August 2024, Yanyun Zhang and Xiaoying Wu 24-1506 (ANUB1892).

#### Verseghya thysanophora

Taxon classificationFungiPertusarialesPertusariaceae

(R.C. Harris) S.Y. Kondr., Lőkös, Farkas & Hur

724535D0-A334-5125-9A14-266A47426335

Fig. 5A–G

≡ Lecanora thysanophora R.C. Harris, in Harris, Brodo and Tønsberg, Bryologist 103(4): 790 (2000) (Basionym).
*= Verseghya klarae* S.Y. Kondr., Lőkös & Hur, Lichens from New and noteworthy lichen-forming and lichenicolous fungi 4: 105–111 (2016). Type: Korea, Gangwon-do, ca. 810 m alt., on bark, 2015, Lőkös, L. 151023 (KoLRI–Holotype).

##### Diagnostic characters.

Thallus leprose, thin, continuous or rimose to verrucose areolate when apothecia present; prothallus white, fibrous, often presenting blackish pigments, 0.5–1.55 mm wide, with hyphae aggregated into distinct radiating strands, hyphae 2.5–5.0 µm wide; upper surface pale green to dark olivaceous green, or pale yellow; soredia fine, rounded, covering the thallus, white or blue-green, [15.0]–(18.9)–24.0–(29.1)–[30.0] µm in diameter (n = 34), non-corticate, often aggregated in elongate to rounded consoredia, up to 80 µm in diameter (Harris et al. 2000). Apothecia lecanorine, rounded, sparse to slightly aggregated, adnate, 0.7–2.2 mm in diameter; disc plane to concave, pale beige to yellowish-brown, with faintly or heavily white pruinose; margin thick and prominent, entire or slightly flexuous, white. Amphithecium with numerous algal cells, interspersed with numerous small crystals, pseudocortex interspersed with fine (POL+, K-sol, N-insol) and coarse crystals (POL+, K-insol, N-sol); parathecium colorless, without crystals (POL-); epihymenium colorless, with superficial coarse crystals (POL+, K-sol, N-sol) or absent, hymenium colorless; subhymenium colorless; hypothecium colorless; paraphyses simple, more or less anastomosed, slender, c. 1.5 µm thick, tips not or slightly thickened; asci clavate, *Lecanora*-type, 8-spored; ascospores simple, hyaline, ellipsoid, [13.0]–(16.1)–18.1–(20.1)–[21.0] × [6.5]–(8.0)–9.9–(11.8)–[14.3] µm (n = 35). Conidia filiform, curved, 18–20 × 0.5–0.7 µm (Kondratyuk et al. 2016).

**Figure 5.**
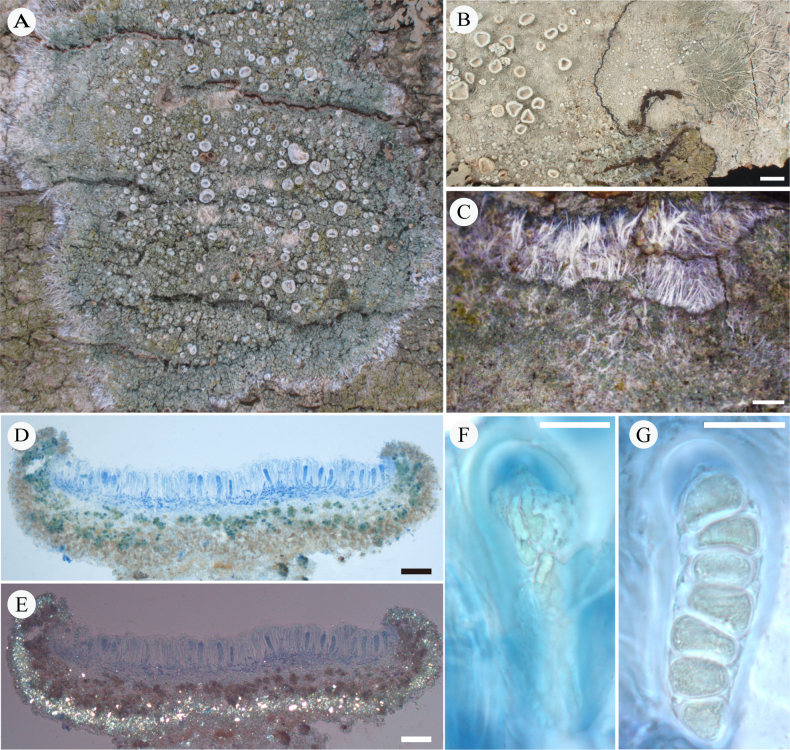
*Verseghya thysanophora* (**A, G** are from FR-0261236, **B, D**–**F** are from FR-0265868, **C** is from ANUB 2353): **A**. Lichen thallus, from South Korea; **B**. Lichen thallus, from Japan; **C**. Lichen thallus, from China; **D**. Vertical sections of apothecia (LCB); **E**. Vertical sections of apothecia in polarized light (LCB); **F**. *Lecanora*-type ascus; **G**. Ascus and ascospores. Scale bars: 2 mm (**B, C**); 100 µm (**D, E**); 10 µm (**F, G**).

##### Notes.

*Lecanora thysanophora* was originally described by Harris et al. (2000) from eastern North America. Later, Kondratyuk et al. (2016, 2019) established a new genus, *Verseghya*, to accommodate this species and described an additional species *V. klarae*, from South Korea. Phylogenetically, the genus *Verseghya* is monophyletic and belongs to the *Verseghya*-*Tylothallia* subclade, which is part of the larger *Verseghya*-*Lecidella*-*Pyrrhospora* clade within the phylogenetic framework of *Lecanoraceae* proposed by Kondratyuk et al. (2016, 2019). Our phylogenetic results are consistent with previous studies, confirming that *Verseghya* forms a well-supported and monophyletic clade (Fig. 1).

However, samples identified as *V. klarae* and *V. thysanophora* from China, the Czech Republic, Japan, Poland, South Korea and the USA cluster together in the phylogenetic tree, forming a distinct and well-supported clade (Fig. 1). No phylogenetic differentiation was detected between the two taxa, indicating that they are conspecific. This conclusion is also supported by morphological and chemical data. Morphologically, both species share a white fibrous prothallus often presenting blackish pigments, lecanorine apothecia, and yellowish-brown discs with faint to heavy, white pruina (Fig. 5). Although in Kondratyuk’s original description, *V. klarae* was reported to possess a *Pertusaria*-type ascus (Kondratyuk et al. 2016), no photographic evidence supported this character, and the specimens examined by Kondratyuk are unavailable for loan. It’s worth noting that all specimens examined in this study, regardless of whether they were collected from South Korea or Japan, exhibited a *Lecanora*-type ascus (Fig. 5F, G). Chemically, both species produce zeorin and usnic acid as major compounds, while *V. thysanophora* may additionally contain atranorin, porphyrilic acid, and “thysanophora unknowns” (Harris et al. 2000; Kondratyuk et al. 2016; Guzow-Krzemińska et al. 2017). Therefore, we propose to reduce *V. klarae* to a synonym of *V. thysanophora*.

##### Specimens examined.

China • Anhui Prov., Lu’an City, Jinzhai County, Dahuanglishuling, 31°10'09"N, 115°50'37"E, 795 m alt., on rotting wood, 2 November 2024, Yanyun Zhang, Yujiao Yin and Yishan Feng 24-1693 (ANUB2353); • Laoshanmiao Village, 31°13'56"N, 115°37'33"E, 726 m alt., on *Cercis gigantean* bark, 1 November 2024, Yanyun Zhang, Yujiao Yin and Yishan Feng 24-1612 (ANUB2272). South Korea • Gangwon-do, Pyeongchang-gun, Jinbu-myeon, Odaesan-ro, Mt. Odae, area around Sangwon Temple, 37°47'14"N, 128°50'00"E, 875 m alt., in mixed gymnosperm-deciduous forest near parking lot, on *Acer picta*, 3 October 2016, Christian Printzen 14122 (FR-0261236); • along the Odae Stream, 37°44'52"N, 128°34'50"E, 710 m alt., on *Quercus mongolica*, 4 October 2016, Christian Printzen 14170 (FR-0261237); • Jeju, Jeju City, Haean-dong, WE trail of Mt. Halla (Eorimok trail), en route from Eorimok to Wisaeorum, 33°23'19"N, 126°29'40"E, 990 m alt., in deciduous forest along riverbed, on bark, 26 September 2016, Christian Printzen 13963 (FR-0261260). Japan • Tochigi Prefecture (Shimotsuke Province), Nikko City administrative region, Nikko National Park, 1.3 km from SSE of Yumoto village, along the trail W of Yukawa stream from Yutaki waterfall to Senjogahara marsh, 36°47'41"N, 139°25'40"E, 1420 m alt., on trunk of *Acer runfinerve*, 28 September 2019, Christian Printzen 15197 (FR-0265868), Christian Printzen 15200 (FR-0265869); • 2 km NNE of Yumoto village (Yumoto Onsen), north facing slope SW of lake Karikomi, 36°49'25"N, 139°25'54"E, 1650–1700 m alt., in dense old growth mixed coniferous/deciduous forest near lakeside, 30 September 2019, Christian Printzen 15462 (FR-0265878).

### Key to the species of *Lecanora* sensu lato in the Anhui Province

**Table:** 

1	Apothecia biatorine, discs variable in color, cream to pink, pale orange or brown	***Zeora symmicta***
–	Apothecia lecanorine, discs brown to reddish-brown or yellow	**2**
2	Prothallus present, fibrous	**3**
–	Prothallus absent or not fibrous	**4**
3	Soredia [22.5]–(27.8)–38.9–(50.0)–[87.5] µm in diameter; prothallus white, fibrous, without blackish pigmentation	***Verseghya megasorediata***
–	Soredia [15.0]–(18.9)–24.0–(29.1)–[30.0] µm in diameter; prothallus white, fibrous, usually with blackish pigmentation	***V. thysanophora***
4	Soredia present	**5**
–	Soredia absent	**7**
5	Thallus margins diffuse, prothallus absent	***Lecanora apicifarinacea***
–	Thallus margins distinct, prothallus present	**6**
6	Soralia immersed in the thallus and arising from the prothallus, rapidly eroding to become irregularly shaped and diffuse, eventually confluent, containing stictic acid as an accessory compound alongside atranorin and zeorin	***L. layana***
–	Soralia marginally small and dispersed, centrally large and aggregated, containing atranorin and zeorin	***L. pseudolayana***
7	Apothecial discs yellowish pruinose	***L. fulvastra***
–	Apothecial discs epruinose or occasionally slightly white pruinose	**8**
8	Apothecia immersed in thallus when young, later sessile	***L. cinereofusca***
–	Apothecia sessile or adnate	**9**
9	Amphithecium with crystals dissolving in K	***L. chionocarpa***
–	Amphithecium with crystals not dissolving in K	**10**
10	Epihymenium without crystals (POL-)	**11**
–	Epihymenium with crystals (POL+)	**13**
11	Apothecial discs plane or moderately concave, asci 8- and 16-spored, producing stictic acid	***L. pseudojaponica***
–	Apothecial discs plane or moderately convex, asci 8-spored	**12**
12	Apothecia 0.3–0.75(–1.1) mm in diameter, discs plane to moderately convex, shiny, margin entire or crenate, producing glabrata unknown -1	***L. glabrata***
–	Apothecia 0.6–2.5 mm in diameter, discs plane, margin entire or flexuous	***L. allophana***
13	Asci 16-spored	***L. anhuiensis***
–	Asci 8-spored	**14**
14	Usnic acid present	***L. subloekoesii***
–	Usnic acid absent	**15**
15	Zeorin present	**16**
–	Zeorin absent	**17**
16	Thallus darker, bluish, olivaceous or pale brownish-gray, discs brown to dark brown, amphithecial cortex present, oil droplets present in apothecial section	***L. baekdudaeganensis***
–	Thallus paler, greenish or yellowish gray, discs reddish-brown, amphithecial cortex indistinct or absent, oil droplets absent	***L. imshaugii***
17	Apothecia 0.3–1.0 mm in diameter, discs reddish-brown to dark brown, prothallus gray or absent, producing pannarin	***L. novae-hollandiae***
–	Apothecia 0.5–2.0 mm in diameter, discs yellowish to reddish brown, prothallus absent, producing gangaleoidin	***L. pseudargentata***

## Supplementary Material

XML Treatment for Lecanora apicifarinacea

XML Treatment for Lecanora pseudolayana

XML Treatment for Verseghya megasorediata

XML Treatment for Verseghya thysanophora

## Additional information

### Artificial Intelligence (AI) use

The authors accept full responsibility for the content of the manuscript, including the disclosure of any use of AI.

No AI tools were used in the preparation of this manuscript.

### Author ORCIDs

L.-J. Li https://orcid.org/0000-0003-1048-1971

Y.-Y. Zhang https://orcid.org/0000-0002-0902-5066

### Data availability

All of the data that support the findings of this study are available in the main text.
